# Intelligent Carbon Dots with Switchable Photo‐Activated Oxidase‐Mimicking Activity and pH Responsive Antioxidant Activity Adaptive to the Wound Microenvironment for Selective Antibacterial Therapy

**DOI:** 10.1002/advs.202406681

**Published:** 2024-09-03

**Authors:** Li He, Zhi Li, Meiqi Gu, Yifei Li, Chengla Yi, Ming Jiang, Xu Yu, Li Xu

**Affiliations:** ^1^ Department of Trauma Surgery, Tongji Hospital, Tongji Medical College Huazhong University of Science and Technology Wuhan 430030 China; ^2^ Tongji School of Pharmacy Huazhong University of Science and Technology Wuhan 430030 China

**Keywords:** antioxidant activity, carbon dot, intelligent antibacterial nanoagent, oxidase‐mimicking activity, selective antibacteria

## Abstract

Intelligent antibacterial agent with controllable activities adaptive to the wound microenvironment is appealing to reduce drug resistance and enhance antibacterial efficiency. In this study, celery is chosen as the carbon source to construct celery‐based carbon dots (CECDs) with double activities, i.e., reactive oxygen species (ROS)‐production and ROS‐clearance activities. The ROS‐production capability of CECDs is dependent on the oxidase (OXD)‐mimicking activity, which is only photo‐activated and thus artificially controlled by light to avoid the production of excess ROS. Meanwhile, the optimal OXD‐mimicking activity occurrs at the pH of 5, close to microenvironmental pH at the bacterial infection site, which will enhance the antibacterial efficacy. On the other hand, CECDs exhibit the antioxidant activity at the neutral or weak alkaline pH, which will assist the healing of the wound. Thus, the conversion of ROS‐production and ROS‐clearance ability of CECDs can be dynamically and intelligently switched automatically with microenvironmental pH at different stages of treatment (from acid to neutral/weak basic). The proposed CECDs exert adorable selective antibacterial activity against Gram‐positive bacteria and satisfactory therapeutic effect on bacteria infected mice. This study paves a new avenue to design the intelligent antibacterial nanoagent sensitive to the infected microenvironmental condition, reducing drug resistance and assisting precise medicine.

## Graphical abstract

Celery‐based carbon dots (CECDs) with double activities, i.e. reactive oxygen species (ROS)‐production and ROS‐clearance activities, are prepared. Photo‐activated oxidase‐mimicking activity and pH responsive antioxidant activity can be intelligently switched adaptive to the wound microenvironment for selective antibacterial therapy.

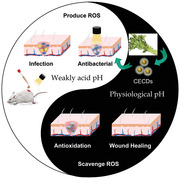

## 1 Introduction

Antibiotics have been world widely used for clinical antimicrobial therapy for about one century. Up to now, antibiotic failure has been becoming a public health problem that cannot be ignored, owing to the drug‐resistance caused by antibiotics abuse or bacterial evolution, immune compromise, sepsis, etc.^[^ ^1^ ^]^ Bacterial infection, especially drug‐resistant one, is a fatal threat to lives and health of human beings. Developing efficient antibacterial agent to avoid drug resistance is arousing high concern of the public and researchers.

Enzymes are mostly protein or RNA ribozyme‐based biocatalysts involved in specific biochemical reactions. Natural enzymes can catalyze H_2_O or O_2_ to produce reactive oxygen species (ROS), which can effectively kill bacteria by destroying cell membrane and membrane proteins. Nevertheless, some defects, like high cost of synthesis and purification, low stability, and harsh catalytic condition, limited their broad applications.^[^ ^2^ ^]^ To address the issue, some alternatives have been developed, e.g. immobilized enzymes, transformed enzymes and nanozymes, etc. Among these, nanozymes, i.e., nanomaterial mimicking natural enzyme activity, are one of the most popular potential alternatives, which are characterized of excellent physical and chemical properties, high stability, adorable biocompatibility, etc.^[^ ^3^ ^]^

Thereinto, with the improved catalytic activity and mild catalytic condition, nanozymes have been becoming one kind of promising antibacterial nanoagent.^[^ ^4^ ^]^ Peroxidase (POD)‐mimicking and oxidase (OXD)‐mimicking nanozymes are predominant for this purpose, which can efficiently produce ROS to oxidize and destroy bacterial cell membrane and biomolecules, and thus effectively inactivate bacteria.^[^ ^2^, ^5^ ^]^ However, POD‐mimicking nanozymes kill bacteria largely by catalyzing H_2_O_2_ to produce ROS, but cannot play an antibacterial role in the absence of H_2_O_2_. The necessity of H_2_O_2_ and uncontrollable activity were the main shortcomings of this kind of nanozyme.^[^ ^1^, ^3^, ^6^ ^]^ As another nanozyme of high concern, OXD‐mimicking nanozyme can catalyze H_2_O or O_2_ to produce ROS without the participation of H_2_O_2_. In terms of necessity of H_2_O_2_ or not, OXD‐mimicking nanozyme was superior to POD‐mimicking one. However, the uncontrollable excessive ROS may be produced to damage normal cells, tissues or biomolecules, causing adverse effect and even reversing therapeutic effect.^[^ ^7^ ^]^ Hence, the nanozyme with controllable activity for antibacterial therapy would be appealing, because controllable antimicrobial activity is activated only when needed,^[^ ^1^, ^7^ ^]^ which would be favorable to decrease the drug resistance.

Unfortunately, in most cases, the activity of nanozymes is all along at a “turn‐on” state and controllability of catalytic activity is seriously lacking. At moment, the environment‐responsive nanozyme is attracting attention for its tunable activity in response to environmental light and/or pHs. For examples, some OXD‐mimicking nanozymes that used light as an artificial switch have been reported in the past few years to achieve controllability, which could mimick OXD activity only under light irradiation to kill bacteria, such as S, N‐doped carbon dots,^[^ ^2d^ ^]^ N‐doped carbon nanodots^[^ ^2a^ ^]^ and Pd‐nano cage,^[^ ^5b^ ^]^ etc. Without irradiation, these nanozymes did not work, which meant that production of ROS could be controllably switched on/off by light irradiation. However, a problem was still present for these nanozymes, i.e., the optimal pH condition for the enzyme activity was quite different from the microenvironmental pH of bacteria infected site. The pHs for optimal enzyme‐like activity of the above‐listed three nanozymes were 3, 3.5, and 4.2, respectively, while the microenvironmental pH at the bacteria infected wound was reported to be ≈5–5.5, and pH would be recovered close to neutral (7.4) when the wound site was healing.^[^ ^1^, ^7^, ^8^ ^]^ Obviously, the microenvironmental pH at the infected site was dynamically changed during the healing process, and disagreed with the optimal pH for maximizing the activity of these nanozymes. As a result, the antibacterial activity of these nanozymes in actual wounds would be more or less compromised, or even completely inhibited. Therefore, it is of great significance to develop nanozymes whose optimal catalytic conditions are close to the microenvironmental pH of bacterial infection site.

Infections occur when foreign invaders multiply within the body, not only inducing damage to cells and tissues through bacterial toxin secreted by bacteria, but also triggering the immune response.^[^ ^1a^ ^]^ Nanozymes could also take effect to reduce the inflammatory response and thus promote wound healing, such as superoxide (SOD)‐ and catalase (CAT)‐mimicking ones.^[^ ^9^ ^]^ Thereinto, SOD‐mimicking nanozymes could reduce inflammation through eliminating O_2_^•−^ from the wound and CAT‐mimicking ones could promote other enzyme activity or reduce inflammation by catalyzing H_2_O_2_ to produce O_2_.^[^ ^9^ ^]^ Actually, both kinds of nanozymes possessed nearly no antibacterial activity themselves, and the antibacterial effect originated from other components, such as loaded metal nanoparticles^[^ ^9a^ ^]^ and antibiotics,^[^ ^9b^ ^]^ etc. For instance, nano‐CeO_2_ with double CAT‐ and POD‐mimicking activities could not only produce ROS to inactivate bacteria, but also eliminate ROS to reduce inflammation.^[^ ^10^ ^]^ But, the contradictory CAT‐ and POD‐mimicking activities were obviously compromised and reduced the therapeutic effect.^[^ ^7a^ ^]^ If the multi‐enzyme‐like activities of nanozymes could be intelligently and dynamically regulated according to microenvironment of infected site, the problem would be addressed.^[^ ^1e^ ^]^ Therefore, it is a creative idea to develop nanozymes with switchable ROS‐producing/clearing activity that can be not only precisely controlled by artificial switch, but also responsive to microenvironment.

In addition to the above‐discussed controllable nanozymes which are supposed to decrease drug resistance, developing selective antibacterial nanoagent is another effective strategy for precise antibacterial purpose. The selective antibacterial activity can not only avoid messing up the delicate balance of microbial flora at the infected site, but also reduce the emergence of drug resistance.^[^ ^1^, ^11^ ^]^ This is also the reason that, in clinical treatment, selection of narrow‐spectrum antibiotics is preferably recommended according to guidelines for clinical use of antibiotics of China. Gram‐positive bacteria were claimed to induce severe infections worldwide and afflicted millions of people every year. According to the statistics, surgical site infections are largely caused by Gram‐positive bacteria.^[^ ^12^ ^]^ Some Gram‐positive bacteria in tumors may enhance chemoresistance, depress the therapeutic effect of chemotherapy drugs, and facilitate the growth of tumor and metastasis. Even worse, as one of the well‐known Gram‐positive bacteria, drug‐resistant *Staphylococcus aureus (S. aureus)* has been regarded as a serious threat on the public health.^[^ ^13^ ^]^ Hence, it is crucial to kill Gram positive bacteria to avoid drug resistance and enhance the public health.

**Scheme 1:**
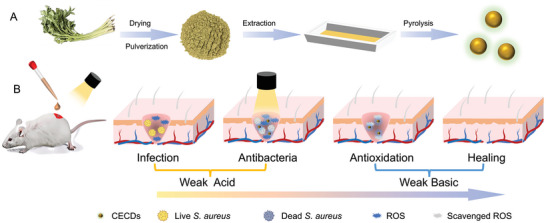
A) Preparation of CECDs. B) CECDs exerting efficacy for antibacterial and promoting wound healing.

In a very recent work, a novel kind of oxygen‐nitrogen functionalized carbon quantum dots (O/N‐CQDs) with photo‐switchable POD/CAT‐like activity was fully studied. It exhibited POD‐like activity without light irradiation, but switched to CAT‐like activity with light irradiation. So, when this O/N‐CQDs was applied as an antibacterial agent, it killed bacteria in the presence of H_2_O_2_. After killing the bacteria, the light was used to activate the CAT activity of O/N‐CQDs to clear the ROS and promote healing the wound. This was an intelligent antibacterial agent responsive to light. However, the conversion of the two enzyme‐like activities was studied under the same pH of 6, ignoring the pH variation at different stages of treatment (from acid to neutral/weak basic). And extra H_2_O_2_ was required for O/N‐CQDs to exert POD‐like activity for antibacterial, which was indispensable but tedious. Additionally, the study only gave the antibacterial performance on *Escherichia coli* (*E. coli*), one of Gram negative bacteria, not mentioning the antibacterial selectivity. Anyway, intelligent nanozyme with switchable activities are encouraging for antibacterial applications. Herein, intelligent carbon dots with switchable visible light‐activated OXD‐mimicking activity and pH responsive antioxidant activity for selective antibacterial therapy were proposed. Celery was chosen as carbon source to construct carbon dots (CECDs) with double activities (**Scheme** **1**A), i.e., the ROS‐producing/clearing activities can be dynamically and intelligently switched in response to pH and light. In weak acidic environment close to the infected wound, CECDs exhibited OXD‐mimicking activity only under light irradiation to produce ROS for selective antibacterial purpose; in neutral/weak basic environment close to the healing wound, CECDs could eliminate ROS to promote healing. The pH range for the conversion of activities of CECDs, from ROS production to clearance, was consistent with the pH variation range at different stages of treatment. Therefore, it is feasible to switch the activities of ROS production to clearance autonomously with the wound healing from acidic to physiological pH, thereby providing an intelligent therapeutic strategy that is adaptive to the healing development of bacterial infection (Scheme 1B). The synergistic effect combining controllability and selectivity of antibacterial activities is promising for antibacterial therapy and reducing the emergence of drug resistance.

## 2 Results and Discussion

### 2.1 Preparation of CECDs

In our study, the biomass‐derived carbon dots (from celery) were prepared. Different to the chemical carbon source with one or more definite compositions, this biomass carbon source contains a great many components which is deemed to be much more complex. As illustrated in Figure S1A (Supporting Information), the celery extraction solution exhibited extremely weak photo‐activated OXD‐mimicking activity, which may be related to some photosensitive compounds in celery, like furanocoumarins.^[^ ^14^ ^]^ However, CECDs did much better, which were supposed to retain some functionalities of celery and thus the photosensitivity as carbon dots could inherit some functionalities from the carbon source.^[^ ^2a^ ^]^ The enhanced activity might be associated with carbonization degree or formation of graphite core of CECDs.

As pyrolysis temperature was influential to the carbonization status of the final material, it was optimized using photo‐activated OXD‐mimicking activity as the criterion by the 3,3ʹ,5,5ʹ‐tetramethylbenzidine (TMB) assay. The stronger absorbance of the oxidized TMB (ox‐TMB) implied stronger photo‐activated OXD‐mimicking activity of the materials, as more TMB was catalytically oxidized. As displayed in Figure S1B (Supporting Information), CECDs pyrolyzed under 270 °C possessed the best photo‐activated OXD‐mimicking activity, which might be related to the nucleation rate.^[^ ^7a^ ^]^ When the temperature was lower than 270 °C, the nucleation rate of CECDs was low, resulting in low catalytic activity. When the temperature was higher than 270 °C, the graphite network of CDs may be destroyed and/or the surface groups were reduced, resulting in weakened activity.^[^ ^6b^ ^]^ Hence, 270 °C was the optimal pyrolysis temperature.

### 2.2 Characterization of CECDs

**Figure 1:**
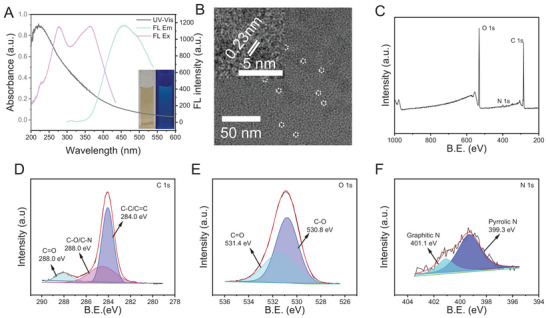
Characterization of CECDs. A) Fluorescence and UV–vis spectra of CECDs aqueous dispersion. Insert: CECDs aqueous dispersion under visible light (left) and UV light (λ = 365 nm) (right). B) TEM and high‐resolution TEM images (inset) of CECDs. C) Survey scan XPS spectrum of CECDs. High resolution XPS spectra of D) C 1 s, E) O 1 s, and F) N 1 s for CECDs.

CECDs were characterized by fluorescence, UV–vis absorbance and X‐ray photoelectron spectra (XPS), and transmission electron microscopic (TEM) observations. The CECDs aqueous dispersion was light brown in bright field and emitted blue fluorescence under UV light (λ = 365 nm) with the maximum excitation wavelength ($\lambda_{\mathrm{ex}}^{\max}$) of 275 and 365 nm and maximum emission wavelength ($\lambda_{\mathrm{em}}^{\max}$) of 455 nm (**Figure** **1**A). An apparent absorption peak at ≈220 nm marked the π→π^*^ electronic transition intrinsic to the aromatic *sp* hybridized region,^[^ ^6^, ^7^ ^]^ which might be originated from the graphitic core of the CECDs. The TEM images disclosed that CECDs appeared as the highly dispersed quasi‐spherical morphology and possessed classical graphite‐carbon lattice structure with a spacing of 0.23 nm (Figure 1B).

XPS measurement was further carried out to investigate the functional groups of CECDs. The survey XPS spectrum confirmed the presence of a few of N in addition to a lot of C and O. In details, the peaks at 288.0 eV (C 1s), 531.4 eV (O 1s), 399.3 eV (N 1s), and 401.1 eV (N 1s) indicated the presence of C = O, pyrrolic N and graphitic N in CECDs (Figure 1D–F), which may be crucial functional groups contributing to OXD‐mimicking activity of carbon‐based nanomaterials.^[^ ^2^, ^3^, ^5^ ^]^

### 2.3 Photo‐Activated OXD‐Mimicking Activity of CECDs

**Figure 2:**
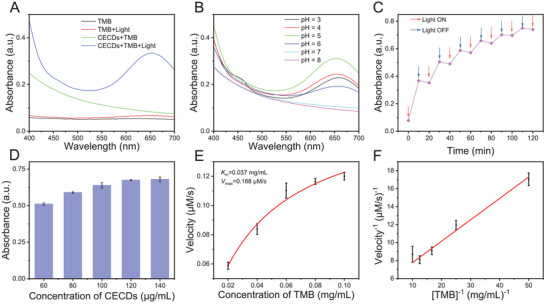
Photo‐activated OXD‐mimicking activity of CECDs. A) UV–vis absorption spectra of CECDs (60 µg mL^−1^) and TMB solution (0.05 mg mL^−1^) as indicated at pH 5.0. B) UV–vis absorption spectra of CECDs (60 µg mL^−1^) and TMB solution (0.05 mg mL^−1^) after light irradiation at different pHs. C) Staircase‐like behavior of the catalytic ability of CECDs with the successive on (red arrows) and off (blue arrows) states of the LED light. D) The effect of CECDs concentration on the OXD‐mimicking activity. E) Steady‐state kinetics assays of CECDs and F) Line‐Burk plot of CECDs under light irradiation. (n = 3 independent samples, data are presented as the mean ± standard deviation (s.d.)).

As discussed above (Section 2.1), the prepared CECDs exerted preferable photo‐activated OXD‐mimicking activity, and herein, this activity was explored in detail. As compared in **Figure** **2**A, only both under irradiation and in the presence of CECDs, TMB can be rapidly oxidized to blue ox‐TMB with a strong characteristic absorbance at 652 nm. In our case, H_2_O_2_ was unnecessary for the oxidation of TMB. As it has been well documented that oxidation of TMB could occur with the assistance of OXD in the absence of H_2_O_2_,^[^ ^2^, ^3^ ^]^ CECDs were deemed to possess OXD‐mimicking activity, but this activity was photosensitive and only activated by the light.

Similar to natural OXD, the pH, enzyme dose, and illumination time may affect the OXD‐mimicking activity of CECDs, and these factors were herein investigated. As for pH, CECDs showed favorable photo‐activated OXD‐mimicking activity when pH ≤ 6, while nearly no activity at pH ≥ 7 (Figure 2B). More importantly, CECDs performed the best catalytic activity at the pH of 5.0, which was close to the microenvironmental pH of bacterial infected site,^[^ ^1^, ^6^, ^8^ ^]^ indicating that CECDs would be suitable to treat bacterial infected wounds. As presented in Figure 2C, when the light irradiation was alternatively switched on or off, the absorbance at 652 nm behaved a staircase‐like response, further demonstrating that OXD‐mimicking activity of CECDs could be only activated by light, and agreed well with the result in Figure 2A. It was worth noting that there was a slight decrease of absorbance at 652 nm within 10 min after light was turned off, possibly suggesting the reducibility of the CECDs (as discussed in Section 2.4).

In order to investigate the kinetics of the CECDs‐catalyzed reaction, the concentration of CECDs was optimized in the TMB assay (Figure 2D). Obviously, the oxidation of TMB by CECDs was concentration‐dependent, as the absorbance at 652 nm strengthened with enhancing CECDs concentration and reached maximum at 120 µg mL^−1^, which was adopted for the following experiments. Both the typical Michaelis–Menten equation and Line‐Burk model were used to fit the kinetics of CECDs within the tested concentration range (Figure 2E,F). The characteristic parameters *K*_m_ and *V*_max_ of the enzyme kinetics (pH 5.0) were 0.037 mg mL^−1^ (0.15 mm) and 0.168 µm s^−1^, respectively. Generally, the smaller *K*_m_ value means that less substrate is required to reach the maximum velocity and a greater affinity between enzyme and substrate. The *K*_m_ of CECDs was 0.15 mm, which was comparable or smaller than most of the reported photo‐activated OXD‐mimicking nanozymes^[^ ^2^, ^5^ ^]^ (Table S1, Supporting Information).

From the above analysis, CECDs not only had optimal OXD activity at the pH of 5.0, which was close to the microenvironmental pH at the bacterial infection site, but also exerted satisfactory enzyme activity in terms of suitable kinetic constants (*V*_max_ and *K*_m_), which would be appreciated for treating bacterial infection.

Furthermore, the active species capture experiments were conducted to investigate the mechanism of the photo‐activated OXD‐mimicking activity of CECDs. The scavengers, including *p*‐benzoquinone (*p*‐BQ), methanol (MeOH), and acridine orange (AO), were adopted to test O_2_^•−^, •OH and h^+^, respectively. Figure S2A (Supporting Information) revealed that the presence of *p*‐BQ pronouncedly suppressed the oxidation of TMB, indicating the predominant formation of O_2_^•−^, but less of •OH and h^+^. Furthermore, the electron spin (paramagnetic) resonance spectrometer (EPR) was used to test the occurrence of O_2_^•−^ under different pHs and irradiation conditions. As depicted in Figure S2B (Supporting Information), the distinctive O_2_^•−^ signal (red line) was remarkably identified with irradiation at pH 5.0, while pure 5,5‐dimethyl‐1‐pyrroline *N*‐oxide (DMPO) in CECDs was EPR silent (black line) without light irradiation under other studied conditions. This result demonstrated that O_2_^•−^ was the main type of ROS produced by CECDs, but the light irradiation and acidic environment was necessary.

### 2.4 Free Radicals Scavenging Activity of CECDs

Immune response of autoimmune system triggered by infections would produce excess ROS, which could hinder wound healing and even aggravate symptoms. Therefore, clearing ROS at the wound site is an effective approach to reduce inflammation and promote the wound healing.^[^ ^15^ ^]^

**Figure 3:**
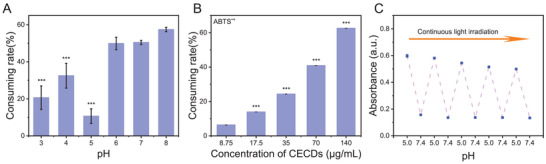
The ROS‐clearance activity of CECDs. A) Consuming rate of ABTS^•+^ at different pHs by CECDs (120 µg mL^−1^). B) Consuming rate of ABTS^•+^ by CECDs with different concentrations. C) The dynamic change of activity of CECDs with the switched pHs. (n = 3 independent samples, data are presented as the mean ± s.d., one‐way analysis of variance (ANOVA), ^***^ *p* < 0.001).

As disclosed above (Section 2.3), CECDs could exert potential reducibility when the light was switched off (Figure 2C) and the photo‐activated OXD‐mimicking activity was largely dependent on the surrounding pHs (Figure 2B). Herein, the antioxidant activity of CECDs was investigated by the 2,2′‐azinobis‐(3‐ethylbenzthiazoline‐6‐sulphonate) (ABTS) scavenging experiment. As displayed in **Figure** **3**A, CECDs behaved strong free radical scavenging ability when pH ≥ 6, but weak when pH ≤ 5, and the weakest occurring at the pH of 5.0. It can be seen that the free radical scavenging ability of CECDs was related to the surrounding pHs and the ROS‐clearance ability was exactly opposite ROS‐production capability under light irradiation (Figure 2B). Additionally, the ability of CECDs to scavenge free radicals was concentration dependent (Figure 3B), and the EC_50_ (concentration for 50% of maximal effect) was ≈110 µg mL^−1^ at the pH of 7.0. Furthermore, the ROS production/clearance could be dynamically and intelligently switched corresponding to the surrounding pHs in the same reaction system. As illustrated in Figure 3C, under weak acidic condition, TMB was oxidized to ox‐TMB, while ox‐TMB was reduced with the obviously weakened absorbance (*λ_max_* = 652 nm) when pH was adjusted to physiological pH of 7.4 in five cycles, and the oxidation activity only slightly decreased in five cycles. To be noted, continuous light irradiation was supplied in these cycles. The reduction of ox‐TMB still occurred, directly illustrating that the antioxidant activity of CECDs was pH decisive irrespective of light while the OXD‐mimicking activity was only activated at acidic pH by light.

Based on the above discussions in Sections 2.3 and 2.4, CECDs could exert adorable photo‐activated OXD‐mimicking activity in weak acidic environment to produce ROS (especially O_2_^•−^), and favorable reducibility to clear ROS at physiological pH (without irradiation). The former property would be appreciated for antibacterial purpose, while the latter for anti‐inflammatory to promote the wound healing. More importantly, the intelligent conversion of ROS‐production/clearance ability was dynamic, cyclic, and pH dependent. This pH responsiveness for the activity conversion of CECDs was consistent with the pH change during the healing of bacterial infection. The microenvironment of bacterial infected wound was weak acidic, and in this case, the light was imposed to assist CECDs to produce ROS to kill bacteria. When the bacteria were killed and the microenvironment would be changed from weak acid to physiological neutral, CECDs would exert ROS‐clearance ability themselves to accelerate the wound healing. In this way, CECDs would be intelligent to treat bacterial infected wounds.

### 2.5 In Vitro Antibacterial Activity of CECDs

**Figure 4:**
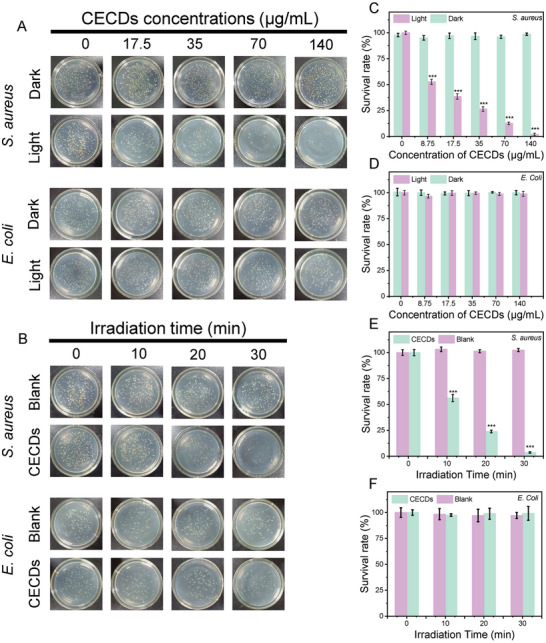
The antibacterial activity of CECDs. A) The photos of colonies of *S. aureus* and *E. coli* treated by CECDs of different concentrations (30 min irradiation). B) The photos of colonies of *S. aureus* and *E. coli* treated by different irradiation time (140 µg mL^−1^ CECDs). Survival rates of C) *S. aureus* and D) *E. coli* treated by CECDs of different concentrations (30 min irradiation). Survival rates of E) *S. aureus* and F) *E. coli* treated by different irradiation time (140 µg mL^−1^ CECDs). (n = 3 independent samples, data are presented as the mean ± s.d., one‐way ANOVA, ^***^ *p* < 0.001).

*S. aureus* and *E. coli* were chosen as the respective model Gram‐positive and Gram‐negative bacteria to study antibacterial activity of CECDs. As illustrated in **Figure** **4**A–C,E, neither of light irradiation nor CECDs alone posed lethality to *S. aureus*, while *S. aureus* was effectively killed under the simultaneous treatment by both light irradiation and CECDs (CECDs + light). This antibacterial activity was dependent on both the dose of CECDs and irradiation time, and the minimum bactericidal concentration (MBC) was 140 µg mL^−1^ at 30 min irradiation. As a contrast, *E. coli* could not be killed under all the studied conditions (Figure 4A,B,D,F). These phenomena indicated that CECDs may possess selective antibacterial activity.

To verify the selective antibacterial activity of CECDs, two other kinds of Gram‐positive (*Staphylococcus epidermidis* and *Streptococcus pneumoniae*) and Gram‐negative (*Bacillus levans* and *Pseudomonas rhodesiae*) bacteria were randomly selected for the antibacterial experiments. As illustrated in Figure S3A,B (Supporting Information), the studied two Gram‐positive bacteria were almost completely killed for the CECDs + light group, while the studied Gram‐negative ones were almost still alive under the same treatment. Thus, CECDs had photo‐activated selective antibacterial activity toward Gram‐positive bacteria. Generally, the cationic surface of the antibacterial agent could assist the antibacterial effect. However, in our case, CECDs were measured to be negative charged under both pH 5.0 and 7.4 (Figure S2C, Supporting Information), which illustrated that surface charge was not responsible for CECDs’ antibacterial ability, in an agreement with the previous report.^[^ ^7a^ ^]^ To test the interaction of CECDs and bacteria, we incubated CECDs (50 µg mL^−1^) with *S. aureus* and *E. coli* of different concentrations for 2 h, and supernatant was collected for fluorescence measurement. As depicted in Figure S5 (Supporting Information), the fluorescence of the supernatant of CECDs with *S. aureus* was obviously weakened, but was almost constant with *E. coli*, reflecting that CECDs interacted with *S. aureus* but not *E. coli*.

Moreover, the live/dead test kit, Calcein acetoxymethyl ester (Calcein‐AM)/propidium iodide (PI), and scanning electron microscopy (SEM) were applied to observe the live/dead state and cell membrane destruction of treated bacteria. As photographed in Figure S4A–H (Supporting Information), the bacteria treated with control, light irradiation or CECDs emitted strong green fluorescence of Calcein but nearly no red fluorescence of PI, indicating that bacteria were alive in these cases. On the contrary, the bacteria treated with CECDs + light emitted very weak green fluorescence of Calcein, but strong red fluorescence of PI, which revealed that bacteria were dead. The destruction of cell membrane and wall were also observed on the CECDs + light treated bacteria (Figure S4L, Supporting Information), while other studied groups appeared complete and smooth surface (Figure S4I–K, Supporting Information), which was consistent with the live/dead staining results. Both the live/dead staining and SEM images evidenced that CECDs could effectively destroy the bacterial cell membrane to kill bacteria.

As discussed above, Gram‐positive bacteria were only effectively killed for the CECDs + light group, implying that antibacterial activity of CECDs could be related to the photo‐activated OXD‐mimicking activity. As demonstrated in Figure S2A,B (Supporting Information), O_2_^•−^ was predominant in the presence of CECDs under irradiation at acidic environment, which would play a crucial role to maintain the ROS balance in bacterial organism, and thus kill bacteria.^[^ ^7a^ ^]^ Additionally, the antibacterial ability of CECDs may be highly related to its nitrogen‐ and oxygen‐ containing structure. As characterized above by XPS and TEM (Figure 1), our CECDs contained pyrrolic N and graphitic N with classical graphite‐carbon lattice structure. It has been well documented that N doping, especially pyrrolic N and graphitic N, contributed much to the enzyme‐like activity and thus the antibacterial properties of carbon dots, as the nitrogen atoms may promote the electron charge transfer and enhance specific surface area, offering more catalytic active sites.^[^ ^2^, ^5^, ^7^ ^]^ Meanwhile, oxygen‐containing functional groups, e.g. –C═O and –O‐H as characterized for our CECDs (Figure 1D–F), have been also demonstrated to provide catalytic and substrate binding sites, elevating the catalytic ability of carbon dots.^[^ ^6a^ ^]^ On the other hand, in consideration that the lack of a physical outer membrane barrier in Gram‐positive bacteria may permit the easier penetration of CECDs^[^ ^1^, ^16^ ^]^ and CECDs interacted with *S. aureus* rather than *E. coli* bacteria, both of these may contribute to the selective antibacterial effect. Moreover, CECDs were generated from celery possessing abundant essential oils, which were claimed to exhibit good inhibition against Gram‐positive bacteria;^[^ ^17^ ^]^ these functional groups of essential oils may be reserved during carbonization, as carbon dots could inherit some functionalities from carbon sources to some degree.^[^ ^2a^ ^]^ Based on these factors, CECDs exerted selective antibacterial ability toward Gram‐positive bacteria. Anyway, in‐depth study is still needed for clarifying underlying antibacterial mechanism.

### 2.6 In Vivo Antibacterial Activity of CECDs

**Figure 5:**
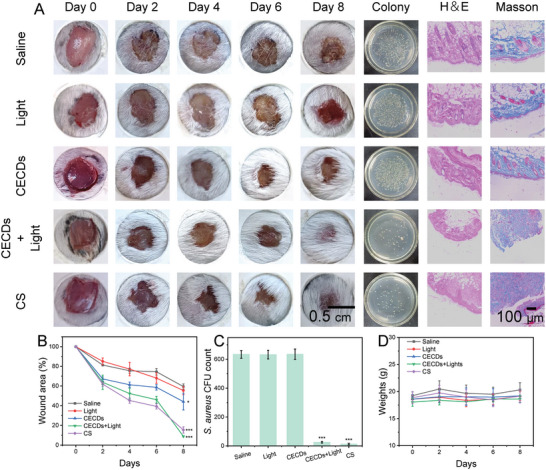
In vivo wound healing by the CECDs. A) Photos of the wound treated with saline, light, CECDs, CECDs+light, and CS at specific time points; photos of bacterial colonies formed on the LB‐agar plates, H&E and Masson‐stained images of wound tissue. B) Relative wound, C) clump count, and D) body weight of mice at specific time points. (n = 5 independent samples, data are presented as the mean ± s.d., one‐way ANOVA, ^*^ *p* < 0.05, ^***^ *p* < 0.001).

A mouse skin *S. aureus* infected wound model was established to evaluate the in vivo antibacterial and promoting wound healing ability of CECDs. Photos of wound healing on the back of mice at different time intervals (**Figure** **5**A) and statistics of wound areas (Figure 5B) disclosed that the wound healing speed was significantly faster in the CECDs + light and Cefquinome sulfate (CS) groups. After one‐day treatment, the infected parts of mice were harvested, homogenized, and coated on lysogeny broth (LB)‐agar plates. Taking the NaCl group as 100%, the bacterial colonies grew on the plate were calculated to be only 3.95 ± 0.78% and 2.00 ± 0.78% in the CS and CECDs + light treatment groups, while abundant bacteria were remarkably survived in the other studied groups (Figure 5A,C). The results demonstrated that CS and CECDs + light groups could both eliminate the bacteria in infected wound and thus reduce the damage of bacteria to infected wound. After 8 days, the healing rates of CECDs + light, CS, CECDs, light and normal saline groups were 91.08 ± 0.56%, 84.64 ± 3.168%, 55.85 ± 8.55%, 44.50 ± 4.09%, and 40.53 ± 2.72%, in this order (Figure 5B), and the former two groups were significantly better than the other studied ones. Additionally, the CECDs + light group exhibited remarkably faster healing rate than that of the CS group, which may be attributed to the ability of CECDs to remove ROS. Meanwhile, the less weight loss of the experimental mice within 8 days (Figure 5D) implied the favorable in vivo biocompatibility of CECDs.

From the H&E and Masson staining results (Figure 5A), the control, light and CECDs group exhibited incomplete skin tissue with obvious inflammatory cell infiltration and uneven collagen fibers. The group treated with CS showed complete skin with a lighter degree of inflammatory cell infiltration and relatively uniform collagen fibers. Ideally, inflammatory infiltrates diminished, hair follicles rejuvenated, and collagen fibers were uniformly distributed in the CECDs + light group. These observations all revealed that, compared to the other studied groups, CECDs could successfully promote infected wound healing, in light of its switchable ROS‐production/clearance activity in response to pHs.

### 2.7 Biocompatibility of CECDs

**Figure 6:**
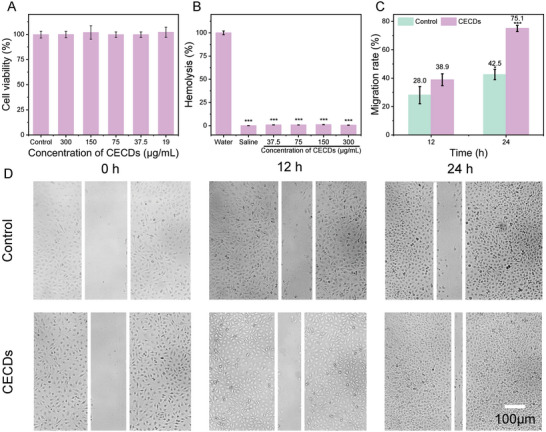
Biocompatibility of CECDs. Cytotoxicity and hemolysis analysis of CECDs at different concentrations to A) HUVECs and B) mouse RBCs, respectively. C) Migration percentage and D) photographs of HUVECs after treatment with CECDs. (n = 3 independent samples, data are presented as the mean ± s.d., one‐way ANOVA, ^*^ *p* < 0.05, ^***^ *p* < 0.001).

**Figure 7:**
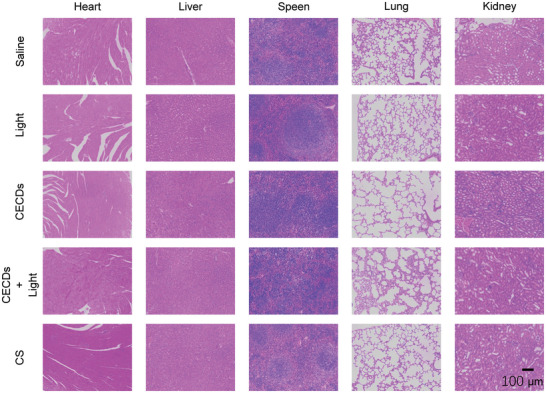
Major organ tissue sections stained with H&E.

Adorable biosafety is an important prerequisite for the application of nanomaterials in vivo. At first, the cell toxicity of CECDs to normal cells (HUVECs) was tested. Almost 100% cell viability was reserved when subjecting to the treatment by CECDs of various concentrations, indicating that CECDs possessed nearly no toxicity to HUVECs even upon the high CECDs concentration up to 300 µg mL^−1^ (2.1‐fold of MBC) (**Figure** **6**A). In addition, CECDs had no obvious hemolysis to red blood cells (RBCs) with the treatment by CECDs of various concentrations (Figure 6B). Furthermore, HUVECs were mechanically scratched to stimulate a wound and then incubated with CECDs for 24 h to evaluate its healing ability (Figure 6C,D). Obviously, CECDs promoted the wound healing most pronouncedly, and the migration rate reached up 38.9% at 12 h, while only 28.0% for the control. With the prolonged treatment, the migration rate was elevated up to 75.1% for the CECDs, almost about twofold of the control (42.5%), indicating that CECDs facilitated the migration of HUVECs, which may add extra benefit to accelerate wound healing. Also, the H&E staining images of the main organs of the treated mice revealed that CECDs exhibited nearly no toxicity to the tissues and organs (**Figure** **7**). From these observations, it can be concluded that CECDs possessed high biocompatibility, supporting its potential for clinical applications.

## 3 Conclusion

Using celery as carbon source, a kind of carbon dots (CECDs) was prepared with switchable ROS‐production and ROS‐clearance activity. CECDs showed photo‐activated OXD‐mimicking activity in weak acidic environment to produce ROS for antibacterial purpose, and exerted antioxidant activity around physiological pH to clear ROS for promoting the wound healing. Moreover, this contradictory ROS‐production/clearance activity could be dynamically and intelligently switched with the microenvironmental pH at different stages of treatment. Through in vitro and in vivo antibacterial experiments, CECDs exhibited favorable selective antibacterial activity against Gram‐positive bacteria, and effectively eliminated bacteria and excess ROS from infected wound, thus promoting wound healing. Additionally, CECDs possessed high biocompatibility without obvious toxicity on cells, tissues, and organs, and hemolysis, while promoting the cell migration. This study developed an intelligent carbon‐based antibacterial nanozyme using the traditional Chinese herb celery for the first time, and provided a new avenue for the design of intelligent antibacterial nanoagent.

## 4 Experimental Section

### Materials

Ethanol (EtOH), NaCl, acetic acid (HOAc), sodium acetate (NaOAc), AO, MeOH, and *p*‐BQ were purchased from Sinopharm Chemical Reagent Co., Ltd. (Shanghai, China). ABTS and TMB were bought from Aladdin Chemical Co. Ltd. (Shanghai, China). Calcein‐AM/PI reagent test kits were bought from Beyotime Biotechnology Co., Ltd. (Shanghai, China). CS was obtained from Yuanye Biotechnology Co., Ltd. (Shanghai, China). Agar powder, yeast extract, and tryptone were commercially available from Sangon Biotech Co., Ltd. (Shanghai, China).

### Apparatus

TEM images were observed by a JEM‐2100 TEM transmission electron microscope (JEOL, Japan). XPS spectra were obtained with an Escalab 250Xi spectrometer (Thermo, USA). The fluorescence and UV–vis absorbance spectra were measured by an F‐4600 fluorescence spectrophotometer (Hitachi, Japan) and a UV‐1800 UV–vis spectrophotometer (Shimadzu, Japan), respectively. A confocal laser scanning microscopy (CLSM, Nikon, Japan) was applied to take confocal photos. SEM images were taken by a field emission SEM (MIRA 3, TESCAN Brno, s.r.o., Russia). The O_2_^•−^ generated by CECDs was studied by an A200 Bruker EPR (Bruker, Germany).

### Preparation of CECDs

The fresh celery (*Apium graveolens L*.), bought from local market (Wuhan, China), was washed and dried in an oven at 70 °C, and crushed into powder with a grinder. Dried celery powder (1 g) was extracted in 20 mL 50% EtOH with sonication for 1 h, and then filtrated to obtain the celery extract. The celery extract (3 mL) was added to a porcelain boat and calcinated in a muffle furnace with heating at a rate of 5 °C min^−1^ from room temperature to 270 °C and maintained at this temperature for 2 h. After natural cooling, 2 mL deionized water was added to the porcelain boat with sonication for 15 min. The obtained turbid solution was centrifuged at 10 000 rpm for 5 min, and the supernatant was collected for dialysis with a 10 000 Da dialysis bag for 48 h. Deionized water was used as the external dialysis solution, and renewed every 12 h. Finally, the solution in the dialysis bag was the CECD aqueous dispersion.

### The TMB Assay

The photo‐activated OXD‐mimicking activity of CECDs was studied by the TMB assay in the absence of H_2_O_2_. Typically, 0.1 mg mL^−1^ of TMB and 70 µg mL^−1^ CECDs were mixed in HAcO‐NaAcO buffer solution (0.01 m, pH 5.0), and then subjected to an LED flat panel luminary (λ ≥ 420 nm, 100 W) irradiation to trigger the oxidation of TMB catalyzed by CECDs. After 10 min irradiation, the absorbance at 652 nm of the mixture was determined. The effect of pH, irradiation time, and CECDs concentration were investigated.

The kinetics of photo‐activated OXD‐mimicking activity was measured through the same procedure by the addition of TMB of different concentrations (0.02–0.1 mg mL^−1^). Michaelis–Menten kinetics equation (Equation (1)) was applied to fit the relationship between the enzymatic reaction velocity and TMB concentrations.

(1) $$V=\frac{V_{\max}\times \left[S\right]}{K_{m}+\left[S\right]}$$

where *V* and *V*_max_ represent the initial and maximal reaction velocity, respectively. [*S*] and *K*_m_ represent TMB concentration and Michaelis constant, respectively. *V* was obtained from the ratio of concentration of ox‐TMB, which was calculated from its absorbance by the Lambert–Beer law, to reaction time (the molar absorption coefficient of ox‐TMB is 39000/(M•cm)). *V*_max_ and *K*_m_ values can also be fitted by Lineweaver Burk's double inverse method (Equation (2)).

(2) $$\;\frac{1}{V}=\frac{K_{m}}{V_{\max}}\;\times \frac{1}{\left[S\right]}+\frac{1}{V_{\max}}$$

### Active Species Capture Experiments

Three kinds of individual ROS scavengers, i.e., 1 mm *p*‐BQ (O_2_^•−^), 1 mm AO (h^+^), and 10 mm MeOH (•OH), were separately added into the above TMB assay system. The TMB assay system without the addition of scavengers was blank, the absorbance of which (*λ* = 652 nm) was normalized as 100%. The production of O_2_^•−^ was further confirmed by EPR using DMPO as a spin trap agent. The spectra and data were recorded with a xenon light source irradiation for 0 min (dark) and 10 min (light).

### The ABTS Assay

The ABTS assay was applied to evaluate the scavenging ability of CECDs against total free radicals. First, 38.4 mg ABTS and 6.59 mg K_2_S_2_O_8_ were dissolved in 10 mL H_2_O and stored in dark over 12 h to produce ABTS^•+^. The mixed solution was diluted to the desirable concentration (with the absorbance at 734 nm ≈0.6–0.9) during use. After adding specific concentrations of CECDs (experimental group) or the same volume of deionized water (control group), the absorbances at 734 nm were determined by a microplate reader, and the consuming rates were calculated according to Equation (3).

(3) $$\mathrm{Consuming}\;\mathrm{rate}\;(\%)\;=\frac{\left[\left(A_{\mathrm{CG}}-A_{C_{0}})-(A_{\mathrm{EG}}-A_{E_{0}}\right)\right]}{A_{\mathrm{CG}}-A_{C_{0}}}\;\times 100\%$$

where A_CG_, A_C0_, A_EG_, and A_E0_ represent the absorbance of control group, water, experimental group, and CECDs of the specific corresponding concentration, respectively.

### Antibacterial Experiments

*Staphylococcus aureus*, *Staphylococcus epidermidis*, and *Streptococcus pneumoniae* were selected as the model Gram‐positive bacteria. *E. coli*, *B. levans*, and *P. rhodesiae* were the model Gram‐negative bacteria. Typically, LB medium containing bacteria was incubated at 37 °C overnight to reach the logarithmic growth period, and then diluted by HOAc‐NaOAc buffer (10 mm, pH 5.0) to 1 × 10^6^ colonies forming units (CFUs). This diluted solution was incubated with CECDs of specific concentration (using the same volume of HAcO‐NaAcO buffer as the control) in a 96‐well plate for 15 min. Afterward, the 96‐well plate was exposed to the LED light prior to the subsequent experiments, i.e., plate count, the live/dead assay, and SEM observation.

### Plate Count Method

After irradiation, the bacterial solution was diluted 50 times by HOAc‐NaOAc buffer, and 50 µL of the diluted solution was spread on the LB‐agar plate. Next, these petri dishes were incubated at 37 °C for 18 h to observe the growth of the bacteria and count the numbers. The effects of concentration of CECDs and irradiation time on the antibacterial activity of CECDs were investigated.

### The Live/Dead Assay

The treated bacteria were collected by centrifugation and washed with HOAc‐NaOAc buffer. Bacteria were stained by Calcein‐Am/PI test kit in the dark for 30 min according to the instruction, and then observed by a CLSM. Generally, the live bacteria would show green fluorescence of the calcein and dead bacteria would show red fluorescence of the PI.

### The SEM Observation

The treated bacteria were collected by centrifugation and fixed by 2.5% glutaraldehyde solution at 4 °C overnight. Afterward, the fixed bacteria were washed with HAcO‐NaAcO buffer for three times, and dehydrated gradually by EtOH in the order of 30%, 50%, 70%, 80%, 90%, and 100% for every 10 min. Finally, thus‐treated bacteria were dropped on a small silicon wafer and dried in the air, subjected to the SEM observation.

### Animal Wound Treatment Experiments

Balb/c female mice (7–8 weeks old) were purchased from Hubei Experimental Animal Research Center. Animal experiments were approved by Institutional Animal Care and Use Committee of Tongji Medical College, Huazhong University of Science and Technology (No. SYXK 2021–0057). Using a ring mold with an inner diameter of 8 mm as a template, surgical scissors were used to create a circular skin wound with a diameter of ≈8 mm on the back of each mouse. Thereafter, 50 µL of *S. aureus* (≈10^9^ CFU) was added to each mouse wound with incubation overnight to establish the infected wound model. The mice were randomly divided into five groups (I)–(V), and treated by dropping the corresponding agents and/or light irradiation, i.e., I) normal saline (50 µL), II) light for 30 min, III) CECDs (300 µg mL^−1^, 50 µL), IV) CECDs (300 µg mL^−1^, 50 µL) + light for 30 min, and V) CS (10 mg mL^−1^, 50 µL). The wounds of the mice were photographed daily and the weights were also recorded. After 8 days, the mice were sacrificed. The wound tissues and major organs (heart, liver, spleen, lung, kidney) were taken for H&E and Masson staining and analysis.

### The Cell Counting Kit‐8 (CCK‐8) Assay

The cytotoxicity of CECDs was assessed using the CCK‐8 assay. HUVECs were seeded in a 96‐well plate with a density of 10 000 cells per well and cultured in DMEM medium with 10% FBS at 37 °C in 5% CO_2_ for 24 h. Subsequently, the cell medium was renewed with fresh medium containing CECDs of different concentrations (experimental group) or the same volume of PBS (blank group), with incubation for additional 24 h. After removing the medium, 100 µL PBS containing 10% CCK‐8 was added and incubated at 37 °C for 1 h. Afterward, the absorbance at 450 nm was measured using a microplate reader, and cell viability was calculated using Equation (4).

(4) $$\mathrm{Cell}\;\mathrm{viability}\;(\%)\;=\frac{A_{E}-A_{C}}{A_{B}-A_{C}}\;\times 100\%$$

where A_E_, A_B_, and A_C_ are the absorbance at 450 nm of the experimental group, blank group, and control group without HUVECs and CECDs.

### Cell Migration Experiment

To evaluate the effect of CECDs on cell migration, HUVECs were seeded in a 6‐well plate with a density of 2 × 10^5^ cells per well and cultured in DMEM supplemented with 10% FBS in 5% CO_2_ for 24 h to form the monolayer. A straight scratch was made in the cell monolayer using a 10 µL pipette tip. Afterward, 2 mL fresh medium containing 150 µg mL^−1^ CECDs (experimental group) or PBS of the same volume (control group) was added. The width of the scratch was observed and imaged by a microscope every 12 h. The cell migration rate was determined using Equation (5).

(5) $$\mathrm{The}\;\mathrm{Cell}\;\mathrm{Migration}\;\mathrm{Rate}\;(\%)\;=\;\frac{L_{0}-L_{t}}{L_{0}}\times 100\%$$

where L_0_ and L_t_ mean the width of the scratch at the time point of 0 and t, respectively.

### The Hemolysis Assay

The hemolysis assay was conducted to assess the hemocompatibility of CECDs. Whole blood was harvested from healthy Balb/c mice and centrifuged at 1500 rmp for 10 min to collect the RBCs. The RBCs were then washed three times with 0.9% NaCl and diluted with 0.9% NaCl to the concentration of 2% (v/v). Next, the RBCs suspension was mixed with CECDs of different concentrations (experimental group) and incubated at 37 °C for 2 h. After incubation, the supernatants of various groups collected by centrifugation at 1500 rmp for 10 min were transferred into a 96‐well plate. The absorbance of the supernatants at 540 nm was determined using a microplate reader. Negative and positive control groups were prepared by mixing the RBCs with 0.9% NaCl and deionized water, respectively. The hemolysis rate was calculated using Equation (6).

(6) $$\mathrm{The}\;\mathrm{Hemolysis}\;\mathrm{Rate}\;(\%)\;=\;\frac{A_{M}-A_{N}}{A_{P}-A_{N}}\times 100\%$$

where A_M_, A_N_, and A_P_ are the absorbance value at 540 nm of experimental, negative, and positive groups, respectively.

### Statistical Analysis

N ≥ 3 independent samples for each statistical analysis, data are presented as the mean ± standard deviation (s. d.), one‐way analysis of variance (ANOVA) was performed by Origin 2024, ^*^ *p* < 0.05, ^**^ *p* < 0.01, ^***^ *p* < 0.001.

## Conflict of Interest

The authors declare no conflict of interest.

## Supporting information

Supporting Information

## Data Availability

The data that support the findings of this study are available from the corresponding author upon reasonable request.
