# Supplementary material for: Intelligent Carbon Dots with Switchable Photo‐Activated Oxidase‐Mimicking Activity and pH Responsive Antioxidant Activity Adaptive to the Wound Microenvironment for Selective Antibacterial Therapy
Source: Adv Sci (Weinh). 2024 Sep 3;11(40):2406681. doi: 10.1002/advs.202406681 (PMC11516101; doi:10.1002/advs.202406681)
Supplement: Supplementary file 1 — Supporting Information [file ADVS-11-2406681-s001.docx]

**Intelligent Carbon Dots with Switchable Photo-activated Oxidase-mimicking Activity and pH Responsive Antioxidant Activity Adaptive to the Wound Microenvironment for Selective Antibacterial Therapy**

*Li He^a^, Zhi Li^b^, Meiqi Gu^a^, Yifei Li^a^, Chengla Yi^a,*^, Ming Jiang^b,*^, Xu Yu^b,*^, and Li Xu^b,*^*

**Table S1** The comparison of photo-activated OXD-like activity of CECDs and other reported nanozymes.

| Nanozymes | *K*_m_ (mM) | *V*_max_ (μM/s) | Reference |
| --- | --- | --- | --- |
| N-CNDs | 0.421 | 0.421 | [6] |
| S, N-CDs | 1.18 | 0.0466 | [9] |
| P-CDs | 0.71 | 0.33 | [10] |
| CDs | 0.22 | 0.0274 | [12] |
| TA-NCDs | 0.61 | 1.21 | [13] |
| Pd-Cage | 0.24 | 0.0708 | [14] |
| CECDs | 0.15 | 0.168 | This work |


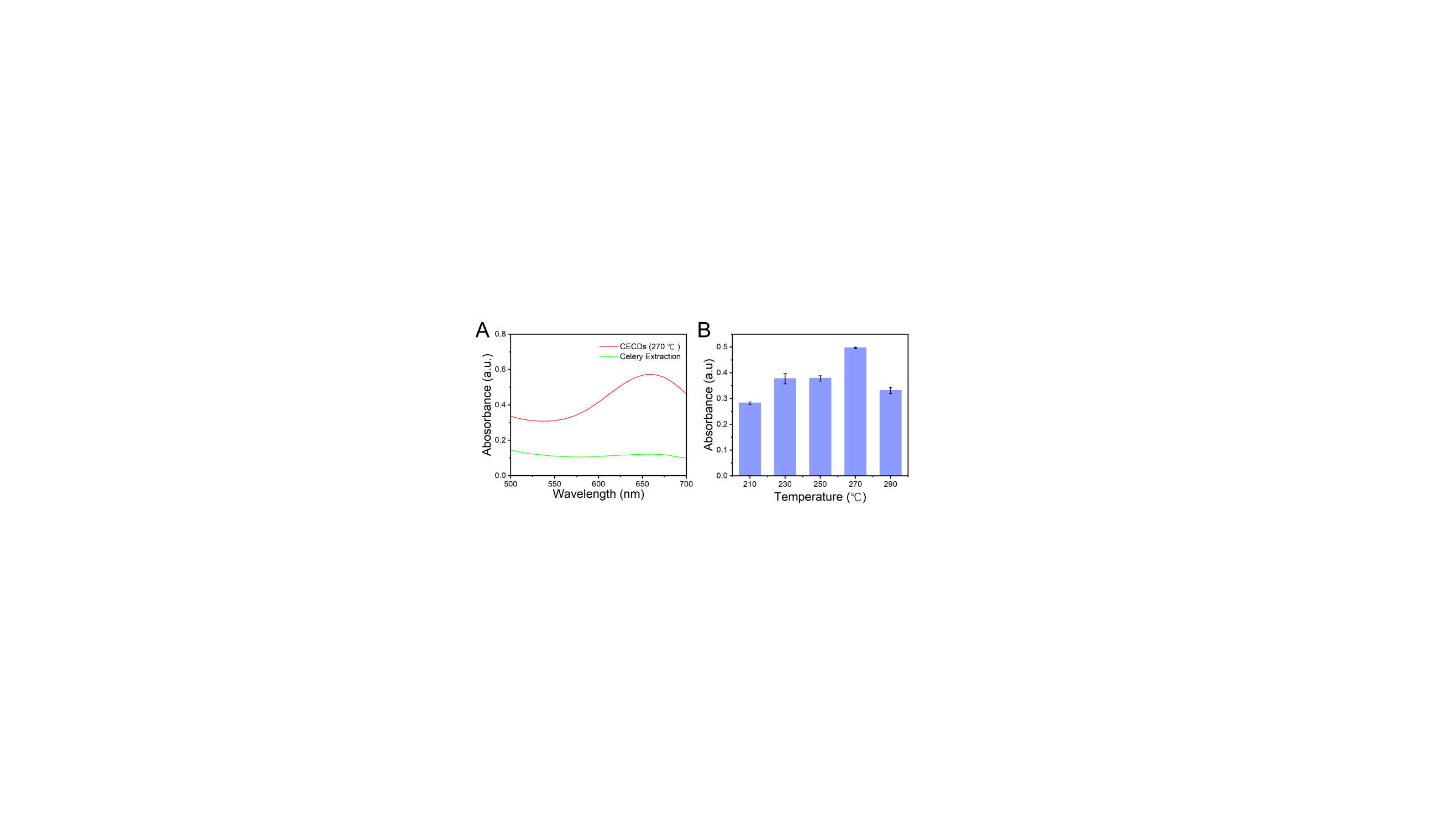


Figure S1 The optimization of preparation of CECDs. (A) The UV spectra of the TMB solution (0.1 mg/mL) in the presence of CECDs (270 ℃) and celery extraction solution (pH 5.0, light irradiation). (B) Absorbance at 652 nm of the TMB solution (0.1 mg/mL) in the presence of CECDs (60 μg/mL) obtained by different pyrolysis temperatures (pH 5.0, light irradiation). (n=3 independent samples, data are presented as the mean ± standard deviation (s.d.), one-way analysis of variance (ANOVA), *p < 0.05 ***p < 0.001).


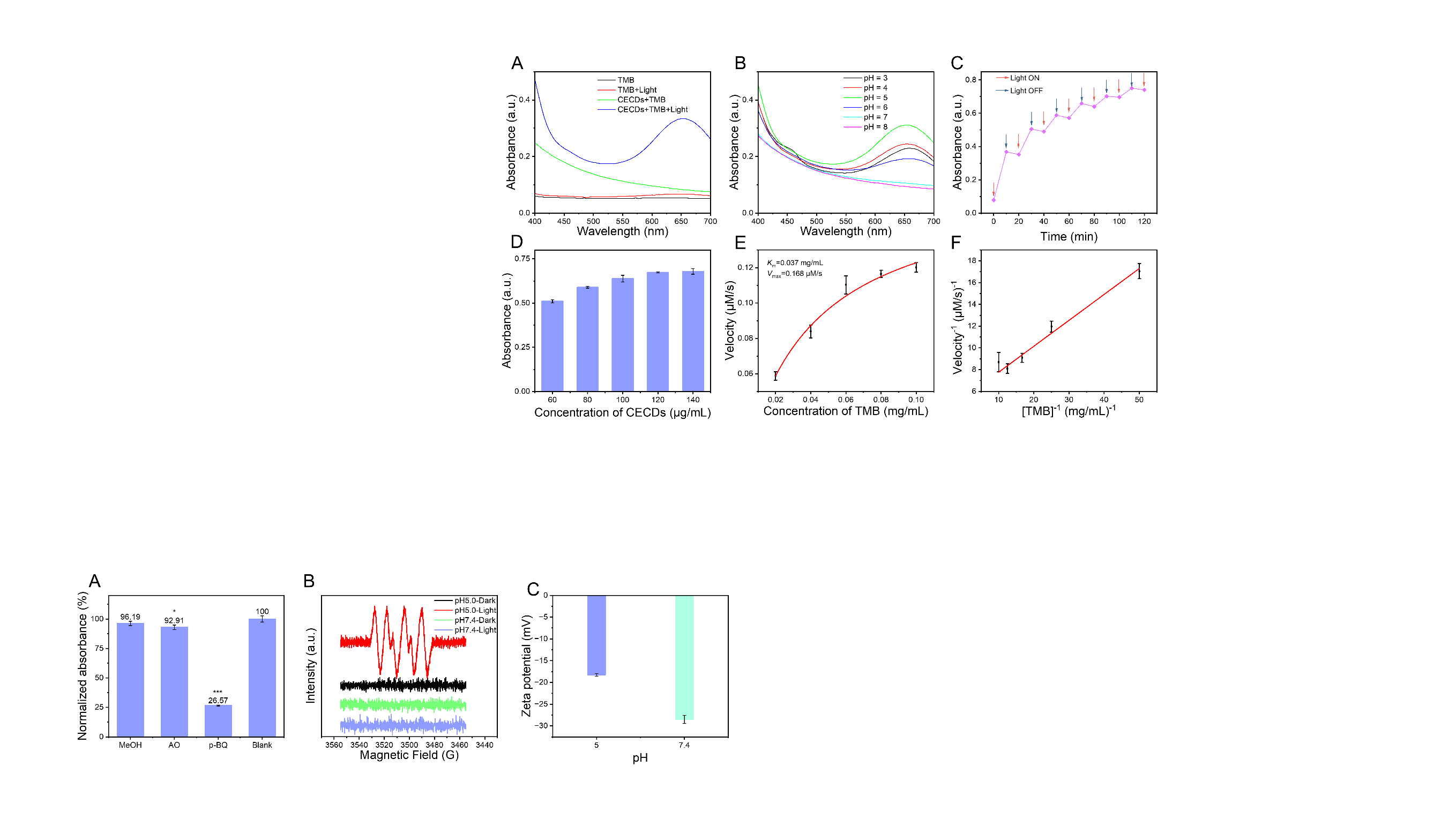


**Figure S2** Mechanism study on the OXD mimicking activity of CECDs. (A) Effect of several scavengers on the oxidation of TMB by CECDs under light irradiation for 10 min. (B) EPR spectra of DMPO + CECDs system at pH 5.0 and 7.4 without and with the light irradiation. (C) Zeta potential of CECDs at pH 5.0 and 7.4. (n=3 independent samples, data are presented as the mean ± s.d., one-way ANOVA, *p < 0.05, ***p < 0.001).


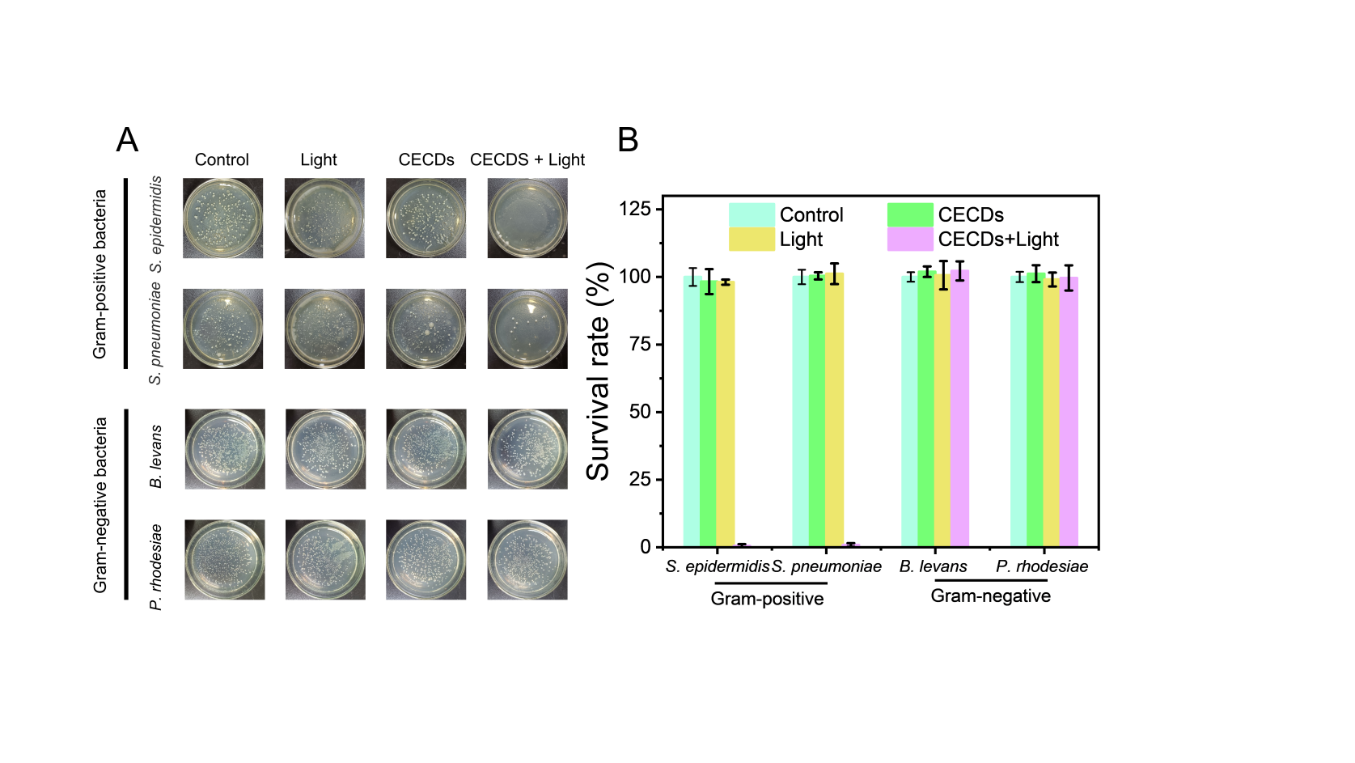


**Figure S3** The selective antibacterial activity of CECDs. (A) Colony photos and (B) survival statistics of Gram-positive (*S. epidermidis* and *S. pneumoniae*) and -negative bacteria (*B. levans* and *P. rhodesiae*) after treatment by 140 μg/mL CECDs and 30 min irradiation. (n=3 independent samples, data are presented as the mean ± s.d., one-way ANOVA, ***p < 0.001).


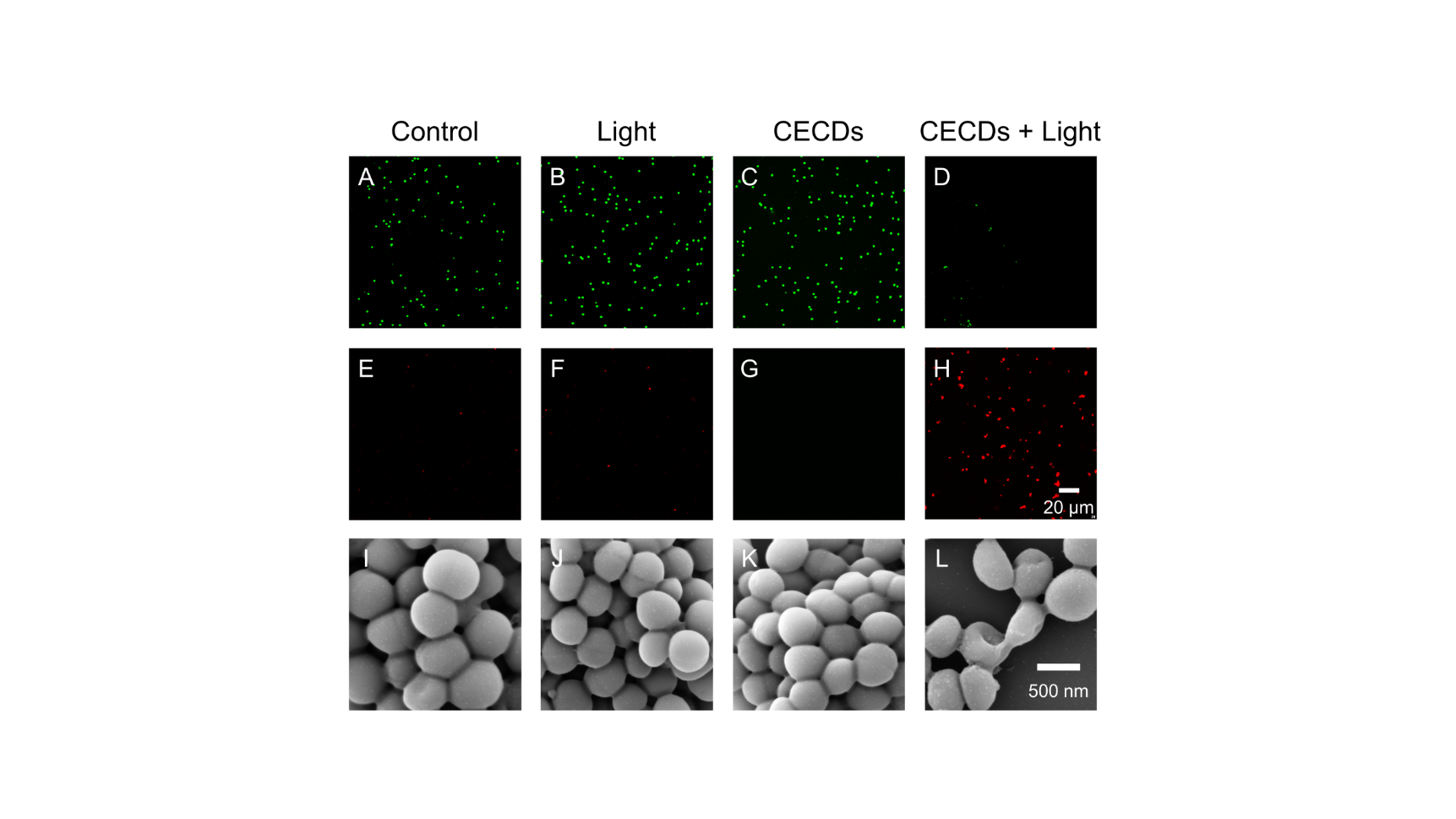


**Figure S4** CLMS images of *S. aureus* by (A and E) untreated, (B and F) light treated alone, (C and G) CECDs treated alone, and (D and H) CECDs + light. SEM images of (I) *S. aureus* without treatment, (J) light treatment alone, (K) CECDs treatment alone, and (L) CECDs +light.

**Figure S5** The interaction of CECDs and bacteria. CECDs (50 μg/mL) were incubated with *S. aureus* and *E. coli* of different concentrations (0, 10^2^, 10^4^, 10^6^, 10^8^ CFU/mL) for 2 h, and the supernatant was collected for fluorescence measurement (λ_ex_=365 nm).
